# Thai health care provider knowledge of neonatal male circumcision in reducing transmission of HIV and other STIs

**DOI:** 10.1186/s12913-015-1182-8

**Published:** 2015-11-25

**Authors:** Kriengkrai Srithanaviboonchai, Boonlure Pruenglampoo, Kanittha Thaikla, Namtip Srirak, Jiraporn Suwanteerangkul, Jiraporn Khorana, Richard M. Grimes, Deanna E. Grimes, Vipa Danthamrongkul, Suchada Paileeklee, Uraiwan Pattanasutnyavong

**Affiliations:** Faculty of Medicine, Chiang Mai University, Chiang Mai, Thailand; Research Institute for Health Sciences, Chiang Mai University, Chiang Mai, Thailand; Division of General Internal Medicine, University of Texas Health Science Center at Houston, Houston, TX USA; Baylor-UT Houston Center for AIDS Research, Houston, TX USA; School of Nursing, University of Texas Health Science Center at Houston, Houston, TX USA; College of Public Health Sciences, Chulalongkorn University, Bangkok, Thailand; Faculty of Medicine, Khon Kaen University, Khon Kaen, Thailand; Faculty of Medicine, Prince of Songkla University, Hat Yai, Songkla Thailand

**Keywords:** Health care provider knowledge, HIV, STIs, Neonatal male circumcision, Thailand

## Abstract

**Background:**

Male circumcision (MC) reduces the risk of female-to-male transmission of HIV and other sexually transmitted infections (STIs). MC has not been practiced as a disease prevention measure in Thailand probably because of low recognition of its benefits among stakeholders. Neonatal male circumcision (NMC) is simpler, safer and cheaper than adult MC. This study aimed to assess Thai health care provider knowledge of benefits implementing NMC in Thailand.

**Methods:**

Multi-stage sampling identified 16 government hospitals to represent various hospital sizes and regions of the country. Researchers administered a fixed choice questionnaire, developed by the research team based on a previous study, to physician administrators, practicing physicians, and nurses whose jobs involved NMC clinical procedures or oversight. The participants reviewed printed educational materials on the benefits of NMC during questionnaire completion. Data were analyzed using descriptive statistics, chi square tests, odds ratios, and logistic regression.

**Results:**

One hundred thirty-three individuals participated in this quantitative study. Only 38 % of the participants agreed that NMC reduced the risk of sexual transmission of HIV while 65 % indicated that they knew that NMC prevented STIs. Most participants recognized the benefits of NMC on hygiene (96 %) as well as cancer prevention (74 %). Major concerns raised were potential trauma to the child, child rights and safety of NMC. After reviewing written information about the benefits of NMC, 59 % of the participants agreed that NMC should be offered in their hospital. Physicians and nurses who had previous experience with circumcising patients of all ages were more reluctant to have NMC performed in their hospital.

**Conclusions:**

A clear policy advocating NMC, thorough preparation of health facilities, and staff training are needed before NMC could be used in Thailand as prevention strategy for HIV and other STIs.

## Background

Three randomized controlled trials have demonstrated that male circumcision (MC) reduces the risk of female-to-male HIV transmission by 51–60 % [1–3]. MC has also been shown to reduce the incidence of other sexually transmitted infections (STIs) [4]. Risk of HPV infection which causes cervical cancer is also lower in female partners of circumcised men than in female partners of uncircumcised men [5]. It has unique superiority over other HIV prevention measures such as condoms, pre exposure prophylaxis (PrEP), and treatment as prevention (TasP). MC is a onetime intervention and the HIV prevention efficacy does not depend on consistent health behaviors related to every act of intercourse or regular pill taking. The World Health Organization has encouraged countries around the world to consider including MC as one of their HIV prevention strategies since 2007 [6]. However, this effective HIV prevention strategy has been under-utilized globally [7]. While adolescent and adult voluntary male circumcision (VMC) may yield almost immediate HIV prevention benefits, lack of demand for the procedure has been a major barrier to achieving circumcision targets [8].

Neonatal male circumcision (NMC) offers advantages over circumcision of adults and has long been used to improve hygiene in some cultures [9]. Srithanaviboonchai and Grimes have previously made the case for NMC as a long-term HIV prevention strategy in Thailand [10]. NMC is simpler, safer, and cheaper than circumcision at later ages [11]. It also has also been shown to reduce the risk of urinary tract infection during first year of life by tenfold [12]. Lack of appropriate analgesia during the procedure may have been a barrier for some parents. However, policies for newborn procedural pain control during NMC has improved during the past 15 years. Dorsal penile nerve block and eutectic mixture of analgesics (EMLA) have been found to be effective in reducing pain during NMC [13]. Thailand provides an excellent environment in which to implement NMC as a means of preventing HIV and other sexually transmitted diseases. Most Thais are Buddhists, and Buddhism does not have any prohibitions against circumcision and low NMC rates are due to low awareness of the procedure and its benefits. Most HIV infections occur through heterosexual contact [14], the transmission risk reduced by MC. Under Thailand’s Universal Coverage health schemes, 99 % of the population have health protection coverage [15]. Thais can easily access health service at no cost or very low cost under these systems. Health care services in Thailand are strong and have been proven to handle HIV interventions with high coverage and good quality. One example of such success is the strong program to prevent mother to child HIV transmission (PMTCT) [16]. Additionally, Thailand now provides antiretroviral therapy to all HIV infected individuals free of charge under the national health insurance system regardless of CD4+ and is the first country in Asia to do so [17]. Thailand has some experience with NMC. A survey of over 700 hospitals found that NMC was performed widely in private hospitals but was rarely performed in government hospitals [18]. Thailand’s strong health care system has meant that almost all women deliver their babies in hospitals after receiving prenatal care [19], so NMC could be introduced to parents during pregnancy and the procedure could be performed soon after birth. This would be consistent with the joint statement issued by the American Academy of Pediatrics and the American College of Obstetricians and Gynecologists, which stated that the health benefits of NMC outweigh the risks and that clinicians should routinely provide unbiased information to parents so that they can decide whether their child should be circumcised [20].

In order to assure that this unbiased information can be provided to parents, it is necessary to determine the degree to which medical and nursing staff are knowledgeable about NMC. This study aimed to ascertain the knowledge level of NMC among Thai health care providers working in government hospitals. Investigators considered it important to restrict the study to personnel who were working in government hospitals for two reasons. First, these are the locations where the majority of births occur. Second, a previous survey revealed that health care providers working in government hospitals were unfamiliar with NMC [18].

## Methods

### Study design

The study utilized a cross-sectional survey of key personnel of varying levels of service in multiple locations. The hospitals were selected to represent the central, northeastern, northern and southern regions of the country and included Pathumthani, Khon Kaen, Chiang Mai, and Songkhla provinces. These provinces were chosen because each had a medical school-affiliated hospital, thus representing all four levels of Thai government hospitals.

Four government hospitals in each province (16 hospitals altogether) were selected as study sites. These included one medical school hospital, one provincial hospital, and two district hospitals in each province. Four medical school hospitals and four provincial hospitals were included by default since there was only one of each type of hospital in each province. For district hospitals, the first one was randomly selected from the ones that were located in the districts adjacent to Amphoe Mueang (the city administrative center of the province) and the second one was randomly selected from the remainder of the hospitals that are located within a certain distance from that city. These hospitals could be classified as either suburban or rural hospitals. The total number of district hospitals for these provinces are 7 for Pathumthani, 20 for Khon Kaen, 23 for Chiang Mai, and 15 for Songkhla.

### Data collection and analysis

The study was conducted between July 2011 and February 2012. Three types of personnel were approached for the study; these were administrators, all of whom were physicians (one per hospital), practicing physicians (2–6 per hospital depending on the size of the hospitals), and nurses whose jobs involve clinical procedures of NMC (5 per hospital). The researchers asked participants to complete a fixed-choice 29-item self-administered paper questionnaire. This questionnaire was developed based on the study team’s previously published critical review on NMC [10] and a previously developed mail survey [18]. The questionnaire included 4 sections: 1) basic demographics; 2) experience in performing circumcision; 3) perception and opinion towards NMC; and 4) opinions with regard to having NMC provided in their facility. Participants were asked to read an information sheet on the benefits of NMC prior to completing the final 2 questions.

Due to small sample size and similar responses on all 12 items of perception and opinion towards NMC, administrators’ responses were combined with physicians. Univariate analysis was conducted to obtain descriptive statistics of all the variables. P values of less than 0.05 was considered statistically significant. Bivariate analyses were performed using Chi-square tests for categorical variables. Factors associated with opposition to implementation of NMC were entered into a multivariate logistic regression model to obtain adjusted odds ratios and 95 % confidence intervals.

### Ethical considerations

Three institutional review boards (IRB) approved the study. These included the IRBs of the Research Institute for Health Sciences, Chiang Mai University; Faculty of Medicine, Khon Khaen University; and Faculty of Medicine, Chiang Mai University. Most hospitals did not have individual IRBs, but instead relied on the ethical approval provided by the IRBs governing the study. Verbal informed consent was obtained prior to the interview.

## Results

### Characteristics of the participants

The overall response rate was 89.9 % (133/148); 68.8 % (11/16) for administrators, 84.6 % (44/52) for practicing physicians, and 97.5 % (78/80) for nurses. Table 1 shows characteristics and experience in performing MC of the participants, with administrators and physicians grouped together due to similarity of responses. In total, 133 health care personnel responded to the questionnaire. Eighteen to twenty nurses responded to the questionnaire in each of the four regions, while 15–17 physicians responded in each of the regions except for Songkhla (southern province) where only 6 responded. Seventy respondents (52.6 %) had previously performed or assisted in an MC procedure, with more physicians experienced than nurses.

**Table 1:** Demographic characteristics and experience in performing circumcision of health care personnel by profession

Characteristics	Physicians (*N* = 55)		Nurses (*N* = 78)		*p*	Total (*N* = 133)	
	n	%	n	%		n	%
Gender							
- Male	35	63.6	2	2.6	<0.001	37	27.8
- Female	20	36.4	76	97.4		96	72.2
Age (mean / SD)	39.8 (10.6)	42.7 (8.6)	0.090	41.5 (9.6)			
- 25 – 44	37	67.3	41	52.6		78	58.6
- 45 – 60	18	32.7	37	47.4		55	41.4
Marital status							
- Married or Cohabiting	34	61.8	54	69.2	0.094	88	66.2
- Single	21	38.2	20	25.6		41	30.8
- Widow/divorced/separated	0	0.0	4	5.1		4	3.0
Length of employment (years)	13.9 (10.5)		19.4 (9.4)		0.232	17.1 (10.2)	
- More than 20	18	32.7	36	46.2		54	40.6
- 20 or less	37	67.3	42	53.8		79	59.4
Ever perform or assist in MC	37	67.3	33	42.3	0.005	70	52.6
Ever perform or assist in NMC	8	14.5	5	6.4	0.120	13	9.8

### Perceived benefits or positive aspects of NMC

Table 2 shows perceived benefits or positive aspects of NMC by health care personnel. Ease of cleaning the penis (95.5 %) received the highest positive response while prevention of HIV infection received the least agreement at 37.6 %. For surgery related items, 66.2 % of the participants thought NMC was safe and 60.2 % thought it was easy to perform. The proportions of favorable responses were similar between physicians and nurses for all benefits (Table 2).

**Table 2:** Profession and benefit perception of NMC

Benefits/positive aspects of NMC	Physicians (*n* = 55)		Nurses (*n* = 78)		*p* =	Total (*N* = 133)	
	n	%	n	%		n	%
Easier to clean penis	51	92.7	76	97.4	0.38	127	95.5
Reduced risk of penile cancer	42	76.4	57	73.1	0.49	99	74.4
Safe to perform	36	65.5	52	66.7	0.92	88	66.2
Reduced risk of STIs	31	56.4	55	70.5	0.10	86	64.7
Reduced risk of cervical cancer	35	63.6	47	60.3	0.70	82	61.7
Easy to perform	31	56.4	49	62.8	0.56	80	60.2
Reduced risk of HIV infection	20	36.4	30	38.5	0.73	50	37.6

### Perceived risks or negative aspects of NMC

The proportion of respondents perceiving these items to be a risk/negative aspect ranged from 9.8 to 86.5 %. More than half of the respondents felt that pain (86.5 %) and inflammation (63.2 %) were possible complications of the surgery. Again, responses on all issues were comparable between physicians and nurses. More details are shown in Table 3.

**Table 3:** Profession and risk perception of NMC

Risks/negative aspects of NMC	Physicians (*n* = 55)		Nurses (*n* = 78)		*p* =	Total (*N* = 133)	
	n	%	n	%		n	%
Pain	46	86.3	69	88.5	0.42	115	86.5
Inflammation	35	63.3	49	62.8	0.92	84	63.2
Violation of child’s rights	23	41.8	35	44.9	0.73	58	43.6
Reduced sexual pleasure	7	12.7	7	9.0	0.47	14	10.5
Inferiority complex as an adult	6	10.9	7	9.0	0.74	13	9.8

### Opinions on whether NMC should be provided at their hospital

After receiving written information about the benefits of NMC, 58.6 % of participants agreed that NMC should be provided in their health facilities. Bnivariate analysis identified the following factors as related to non-agreement to have NMC provided in their health facilities: 1) previous experience with performing or assisting in a MC procedure (OR 4.27, 95 % CI 2.02 – 9.02) and being a physician as opposed to being a nurse (OR 2.23; 95 % CI 1.10 – 4.53). Adjusting for both of these factors in multivariate analysis, the only factor that remained statistically significant was previous experience with performing or assisting in MC (OR 3.82; 95 % CI 1.78 – 8.21) (Table 4).

**Table 4:** Opposition to implementation of NMC service in the hospital

Characteristics	My hospital should not provide NMC service (%)	Crude OR (95 % CI)	Adjusted OR (95 % CI)
Age (years)			
- 45 – 60	34.5	1	a
- 25 – 44	46.2	1.62 (0.80 – 3.31)	
Gender			
- Female	37.5	1	a
- Male	51.4	1.76 (0.82 – 3.78)	
Marital status			
- Single/widow/divorced/separated	40.2	1	a
- Married	42.2	1.09 (0.52 – 2.26)	
Profession			
- Nurse	33.3	1	1
- Physician	52.7	2.23 (1.10 – 4.53)	1.70 (0.80 – 3.62)
Ever performed or assisted in MC?			
- Never	23.8	1	1
- Yes	57.1	4.27 (2.02 – 9.02)	3.82 (1.78 – 8.21)
Length of employment (years)			
- More than 20	33.3	1	a
- 20 or less	46.8	1.76 (0.86 – 3.61)	

^a^not included in multivariate analysis

## Discussion

These results demonstrate the significant barriers that must be overcome if NMC is to be implemented in Thailand. There is significant misinformation or lack of information about the benefits of NMC. The fact that one-third of nurses and physicians did not know that MC reduces the risk of contracting a sexually transmitted infection is worrisome. Even more concerning is that nearly two-thirds of the respondents did not know that MC reduces the chance of acquiring HIV infection. Given the modest experience that the physicians and nurses had with either adult or neonatal circumcision it is not surprising that one-third of the respondents thought NMC to be unsafe and nearly two out of five thought that the procedure was not easy to perform.

The respondents did correctly identify pain and potential inflammation as possible complications following NMC. However, it is not clear why so many of them thought that NMC was a violation of the child’s rights. These perceived negative aspects must have a strong impact because a third of nurses and over half of physicians did not feel that NMC should be an available service in their hospitals, despite receiving the information sheet describing the health-related benefits of NMC. However, it is possible that the information learned in the brief information sheet may have positively changed some opinions. In a nationwide hospital-based survey, it was found that only 27.7 % of 337 Thai nurses felt that NMC should be available in their hospitals. Of the 196 physicians in the survey, 32.7 % thought that their hospital should provide NMC [18]. This suggests that more intensive education might overcome the reluctance to provide the service. The association between prior experience with performing circumcision and not feeling that NMC service should be provided was a surprising finding. This issue needs to be explored further to better understand this phenomenon.

Using NMC as an HIV prevention strategy relies on having a policy in place. Before a policy can be developed, several issues must be resolved. In order for NMC to be implemented in governmental hospitals, the Ministry of Public Health would have to provide the funds for the procedure and training of clinicians on how to perform NMC. There would also need to be funding for training nurses in how to care for newborns following the surgery and how to instruct parents in home care after discharge from the hospital. Also, written instructions for parents will need to be prepared. There are also issues with regard to whether NMC should be provided by all hospitals or only by certain hospitals. If NMC is to be made available throughout Thailand, then medical and nursing school curricula will have to be altered to reflect the change since NMC is not currently a requirement in the curricula.

Scientifically-based education programs for parents will need to be developed so that they can make informed decisions about NMC. Presumably these programs would be developed by the Ministry of Public Health in consultation with clinicians. Funds will need to be provided to develop and test these materials. These are significant policy issues that should be approached with caution until the proper direction is clear. Therefore, we recommend implementing pilot studies of NMC and outcomes throughout the country. The lessons learned from these pilot studies can then be used to develop a plan for nationwide implementation.

The advantages of this study include obtaining information from physicians and nurses who work in all four levels of government hospitals (medical school hospitals, provincial hospitals, and both suburban and rural community hospitals) in all four regions of the country. Data shown in Table 2 also showed a wide range of age and length of work experience of these providers. Limitations include the relatively small sample size and the lack of a robust response from the physicians in the southern region. However, this may not have a large impact on policy issues relating to NMC since the majority of people in the south are Muslims. Boys in this culture are circumcised as pre-adolescents as part of a religious rite and are unlikely to undergo NMC. Since some of the participants and the researchers knew each other, presence of researchers during the completion of questionnaires may have introduced bias or influenced responses. Respondents are volunteers and might not represent the opinions of all health provider staff of their hospitals. And lastly, the results have limited external validity since the contexts regarding MC and NMC are very country specific.

## Conclusion

This study highlights important barriers among health care professionals that need to be overcome if Thailand is to implement NMC. The results suggest that a great deal of education of health professionals is necessary if NMC is likely to become a widespread practice. Further studies of the issues and the resolution of barriers to NMC need to be carried out. There will also have to be strong leadership from the Ministry of Public Health to establish NMC as a national priority. The potential benefits, however, to males and their future female partners are significant.

## References

[1] Auvert B, Taljaard D, Lagarde E, Sobngwi-Tambekou J, Sitta R, Puren A (2005). Randomized, controlled intervention trial of male circumcision for reduction of HIV infection risk: the ANRS 1265 trial. PLoS Med.

[2] Bailey RC, Moses S, Parker CB, Agot K, Maclean I, Krieger JN (2007). Male circumcision for HIV prevention in young men in Kisumu, Kenya: a randomised controlled trial. Lancet.

[3] Gray RH, Kigozi G, Serwadda D, Makumbi F, Watya S, Nalugoda F (2007). Male circumcision for HIV prevention in men in Rakai, Uganda: a randomised trial. Lancet.

[4] Morris BJ, Hankins CA, Tobian AA, Krieger JN, Klausner JD (2014). Does male circumcision protect against sexually transmitted infections? Arguments and meta-analyses to the contrary fail to withstand scrutiny. ISRN Urol.

[5] Wawer MJ, Tobian AA, Kigozi G, Kong X, Gravitt PE, Serwadda D (2011). Effect of circumcision of HIV-negative men on transmission of human papillomavirus to HIV-negative women: a randomised trial in Rakai, Uganda. Lancet.

[6] World Health Organization and the Joint United Nations Program on HIV/AIDS (2007). New data on male circumcision and HIV prevention: policy and programme implications.

[7] Tobian AA, Kacker S, Quinn TC (2014). Male circumcision: a globally relevant but under-utilized method for the prevention of HIV and other sexually transmitted infections. Annu Rev Med.

[8] Ledikwe JH, Nyanga RO, Hagon J, Grignon JS, Mpofu M (2014). Scaling-up voluntary medical male circumcision - what have we learned?. HIV AIDS (Auckl).

[9] AlGhamdi KM, AlHomoudi FA, Khurram H (2014). Skin care: historical and contemporary views. Saudi Pharm J.

[10] Srithanaviboonchai K, Grimes RM (2012). Why Thailand should consider promoting neonatal circumcision?. Southeast Asian J Trop Med Public Health.

[11] World Health Organization (2010). Manual for early infant male circumcision.

[12] Schoen EJ, Colby CJ, Ray GT (2000). Newborn circumcision decreases incidence and costs of urinary tract infections during the first year of life. Pediatrics.

[13] Brady-Fryer B, Wiebe N, Lander JA (2004). Pain relief for neonatal circumcision. Cochrane Database Syst Rev.

[14] Bureau of Epidemiology. HIV situation in Thailand. 2011. http://www.aidsthai.org/en/contents/view/8. Accessed 15 Nov 2014.

[15] World Bank Thailand. Thailand: Sustaining Health Protection for all. http://www.worldbank.org/en/news/feature/2012/08/20/thailand-sustain-health-protection-for-all. Accessed 22 August 2015.

[16] Plipat T, Naiwatanakul T, Rattanasuporn N, Sangwanloy O, Amornwichet P, Teeraratkul A (2007). Reduction in mother-to-child transmission of HIV in Thailand, 2001–2003: results from population-based surveillance in six provinces. AIDS.

[17] Bureau of AIDS, TB, and STI (2014). Thailand national guideline on HIV/AIDS treatment and prevention 2014.

[18] Srithanaviboonchai K, Grimes RM, Suwanteerankul J, Thaikla K, Korana J, Pruenglampoo B (2014). Capability of Thailand to implement newborn male circumcision: a nation-wide survey. AIDS Care.

[19] Kongsri S, Limwattananon S, Sirilak S, Prakongsai P, Tangcharoensathien V (2011). Equity of access to and utilization of reproductive health services in Thailand: national Reproductive Health Survey data, 2006 and 2009. Reprod Health Matters.

[20] American Academy of Pediatrics (2012). Circumcision policy statement. Pediatrics.

